# Prevalence of metabolic syndrome among adult population in India: A systematic review and meta-analysis

**DOI:** 10.1371/journal.pone.0240971

**Published:** 2020-10-19

**Authors:** Yuvaraj Krishnamoorthy, Sathish Rajaa, Sharan Murali, Tanveer Rehman, Jayaprakash Sahoo, Sitanshu Sekhar Kar

**Affiliations:** 1 Department of Preventive and Social Medicine, Jawaharlal Institute of Postgraduate Medical Education and Research (JIPMER), Puducherry, India; 2 Department of Endocrinology, Jawaharlal Institute of Postgraduate Medical Education and Research (JIPMER), Puducherry, India; University of Oxford, UNITED KINGDOM

## Abstract

**Objective:**

This review was done to determine the prevalence of metabolic syndrome (MS) among adult general population in India. We also wanted to find the gender, setting, and region-wide distribution of MS in India.

**Methods:**

We conducted systematic searches in various databases including Medline, ScienceDirect, Cochrane library and Google Scholar from inception until August 2019. We included studies conducted in India reporting the prevalence of MS among adults aged 18 years or more. We used the Newcastle Ottawa scale to assess the quality of included studies. We carried out a meta-analysis with random-effects model and reported pooled prevalence with 95% confidence intervals (CIs). We used the Funnel plot to assess publication biases.

**Results:**

In total, we analysed 113 data from 111 studies with 133,926 participants. Majority of the included studies (76 out of 111) had low risk of bias. We found significant heterogeneity among the included studies (p<0.001). We also found a symmetrical funnel plot indicating an absence of publication bias. The prevalence of MS among adult population in India was 30% (95%CI: 28%-33%). There was a steady increase in the burden across the age groups from 13% (18–29 years group) to 50% (50–59 years). We also found that people living in urban areas (32%; 95%CI: 29%-36%) had higher prevalence when compared to tribal (28%; 95%CI: 21%-36%) or rural adults (22%; 95%CI: 20%-25%). Gender distribution of MS showed that the females had higher prevalence (35%; 95%CI: 31%-38%) when compared to males 26% (95%CI: 22%-29%).

**Conclusion:**

Almost one in three adults in India suffer from MS. Females, people living in urban areas and in northeast region had higher prevalence of MS. Development and implementation of policies and protocols for the screening of MS would enable us in early diagnosis and treatment with special focus towards the vulnerable and high-risk groups.

## Introduction

Disease patterns around the globe are undergoing rapid structural changes over the last three decades, with a sudden increase in the burden of Non-Communicable Diseases (NCDs) and a decreasing trend of communicable diseases [1]. The Global Burden of Disease (GBD) study, brings to light this phenomenon of epidemiological transition in India, with a 62.7% of the total mortality in 2016 contributed by the NCDs. Key elements contributing to the development of these NCDs have been identified and are studied together under the heading of Metabolic Syndrome (MS) [2].

Metabolic syndrome is a constellation of interconnected physiological, biochemical, clinical and metabolic factors that directly increases the risk of cardiovascular diseases, type 2 diabetes mellitus (DM) and all-cause mortality. It is constituted by abdominal obesity, insulin resistance, hypertension, and hyperlipidemia [3]. Various diagnostic criteria have been proposed for quantifying MS. But the most widely used ones are from the International Diabetic Federation (IDF) and the National Cholesterol Education Program Adult Treatment Panel III (NCEP ATP III) [4, 5].

Metabolic syndrome increases the risk of developing type 2 DM and cardiovascular diseases over the next 5 to 10 years by five and two-fold respectively [3]. Furthermore the patients with MS have, an average four-fold increased risk of developing stroke & myocardial infarction and a two-fold risk of dying from a similar event compared with those without MS, regardless of previous history of cardiovascular events [6]. Identifying the individuals with (or) at risk of developing MS would help to inform the probabilities of worse outcomes and thus an urgent need for a promotive, preventive or curative action.

A recent study from the United States reported the prevalence of MS to be around 22.9% [7]. Various population-based studies were conducted in India too, to quantify the same and the results ranged from 10 to 30 percentage [8]. A study conducted in the eleven large urban cities of India during 2006–2010 reported the prevalence of MS as high as 35% [9]. Owing to the behavioural habits, urban population, seem to be the most vulnerable group for developing MS [10]. Prevalence of MS, thus, seems to vary greatly from region to region and from ethnicity to ethnicity.

Even though evidences are being generated on MS from different parts of the country, there is no nationwide pooled estimate to comment on the burden of MS in the Indian subcontinent, which could drive policy action [11]. With almost one third of the total population living in the urban areas, and with 40% of the population aged between 30 and 70 years, understanding the overall prevalence of MS in India becomes essential for predicting the future burden of type 2 DM and cardiovascular diseases [12].

While the NCD care in the country is segregated under the broad headings of Diabetes, and Cardiovascular diseases, MS informs us the need to look at the picture as a whole. Results from this study would, thus, help guide national policy making process to give due importance to prevention and treatment of MS in the community and clinical settings. This could thus be an essential key to keep a check on the ever-increasing burden of the NCD epidemic in the country. Hence, this review was conducted to generate a pooled prevalence estimate for MS in India.

## Methods

### Design and registration

We conducted a systematic review and meta-analysis of cross-sectional studies. We have obtained approval from the JIPMER Scientific Advisory Committee (JSAC). We have also obtained exemption from Institutional Ethics Committee (IEC). We have registered our protocol on the international prospective register of systematic review (PROSPERO). PROSPERO Registration Number is CRD42019147277. We followed the Preferred Reporting Items for Systematic Reviews and Meta-Analyses (PRISMA) checklist for reporting systematic reviews incorporating meta-analyses for reporting our review.

### Eligibility criteria

#### Type of studies

We included studies conducted in India reporting the prevalence of MS for the current review. There was no restriction related to study design, communities (rural/urban) or age groups. We included the studies irrespective of the setting in which the study was conducted (community or facility based or workplace based). Studies reported as full text were included while unpublished data were excluded from the review.

#### Type of participants

We included studies conducted among adults aged 18 years or more. We excluded the studies conducted among specific diseased population.

#### Type of outcome measure

Studies reporting the prevalence of MS and diagnosed it as per the NCEP ATP III or IDF guidelines were included to obtain the pooled prevalence estimate for MS in India.

### Search strategy

We conducted extensive electronic search in the following databases: Medline, ScienceDirect, Cochrane library and Google Scholar. Combination of medical subject heading (MeSH) and free text terms were used for carrying out literature search. The detailed search strategy used to search the Medline database has been reported in **S1 File**. Similar strategies were used in Cochrane Library, ScienceDirect and Google scholar for literature search of published studies. Search was conducted in all the databases from inception of the database to August 2019 with English language restriction for publication. We also checked the reference list of primary studies obtained through electronic search and relevant articles were included in the review and analysis.

### Selection of studies

Two independent investigators (YK and TR) independently performed the literature search and screened the title, abstract and keywords of all the studies identified for possible inclusion in the review. Full text article was obtained for those studies that are found to be relevant. Further screening of abstract and full text of the retrieved articles was done independently by two investigators (YK and TR) to select the studies which satisfy the eligibility criteria of the current review. Any disagreements during the entire selection process between two authors were resolved either through consensus or consultation with third investigator (SR). Quality of the overall review process was monitored by the third investigator (SR).

### Data extraction and management

Primary investigator (YK) extracted the relevant study characteristics for the review from the included studies using the Cochrane Public Health group Data extraction and Assessment Template. Following data were extracted:

#### General information

Author, Study title, Publication year.

#### Methods section

Study design, study period, study setting (community/workplace/facility), community (urban/rural/tribal), geographical region, state, sample size, sampling technique, diagnostic criteria, outcome assessment and statistical tests employed.

#### Outcome section

Mean age, non-response rate and their characteristics, prevalence of MS.

Primary investigator (YK) transferred the obtained data into the software STATA version 14. Data entry was double checked for correct entry by another investigator (SR) through comparison of data presented in review and included study reports.

### Risk of bias assessment in included studies

Two independent authors (SR and SM) assessed the quality of all the included studies using the Newcastle-Ottawa (NO) scale adapted for cross sectional studies [13]. Two criteria (selection and outcome) were used to assess the risk of bias. Following domains were used for assessing the risk of bias under selection criteria: representativeness of the sample, justification of sample size, rate of non-respondents and their characteristics and use of validated measurement tool. Under Outcome criteria, outcome assessment and statistical test employed were used to assess the risk of bias. Each of these outcomes was rated as high (1 point) or low (0 points) based on the quality of evidence and availability of information. Studies scoring more than or equal to 3 points were considered to have high risk of bias.

### Statistical analysis

Meta-analysis was performed with the selected studies using STATA 14.2 (StataCorp, College Station, TX, USA). For each of the studies, standard error was calculated using the reported prevalence of MS and total sample size. “Metaprop” function was used for performing analysis of the current review [14]. To minimize the effect of extremely small or large prevalence on the overall estimate, Freeman Tukey double arc-sine transformation was done to stabilise the variance [14]. Final pooling of data was done with random effects model and reported as proportion with 95% confidence interval.

Evidence of between-study variance due to heterogeneity was assessed through following methods: Chi square test for heterogeneity and I^2^ statistics to quantify the inconsistency. I^2^ less than 25% was mild, 25–75% was moderate and more than 75% was considered as substantial heterogeneity [15]. Sub-group analysis was performed based on age group, gender, geographical region (North/South/West/East/Northeast/Central), study setting (community/workplace/facility), diagnostic criteria, waist circumference measurement, year of publication, quality of studies, representativeness of the sample and urban/rural/tribal communities. Potential covariates for meta-regression were study setting, region, diagnostic criteria, representativeness of the sample, year of publication, mean age and quality of evidence. We performed multivariable meta-regression analysis by including the study level factors with p value less than 0.2 in the univariate model. We performed sensitivity analysis to assess the robustness of the results by excluding the studies conducted exclusively on elderly (≥60 years), studies having high risk of bias or not conducted on a representative sample, and studies conducted before 2007.

Study specific prevalence estimates and pooled estimates were graphically represented through forest plot for both combined and subgroup analysis. Publication bias was assessed and graphically represented by funnel plot; asymmetry of the plot was tested using Egger’s test and p value less than 0.10 was considered as statistically significant publication bias [16].

## Results

### Study selection

**Fig 1:**
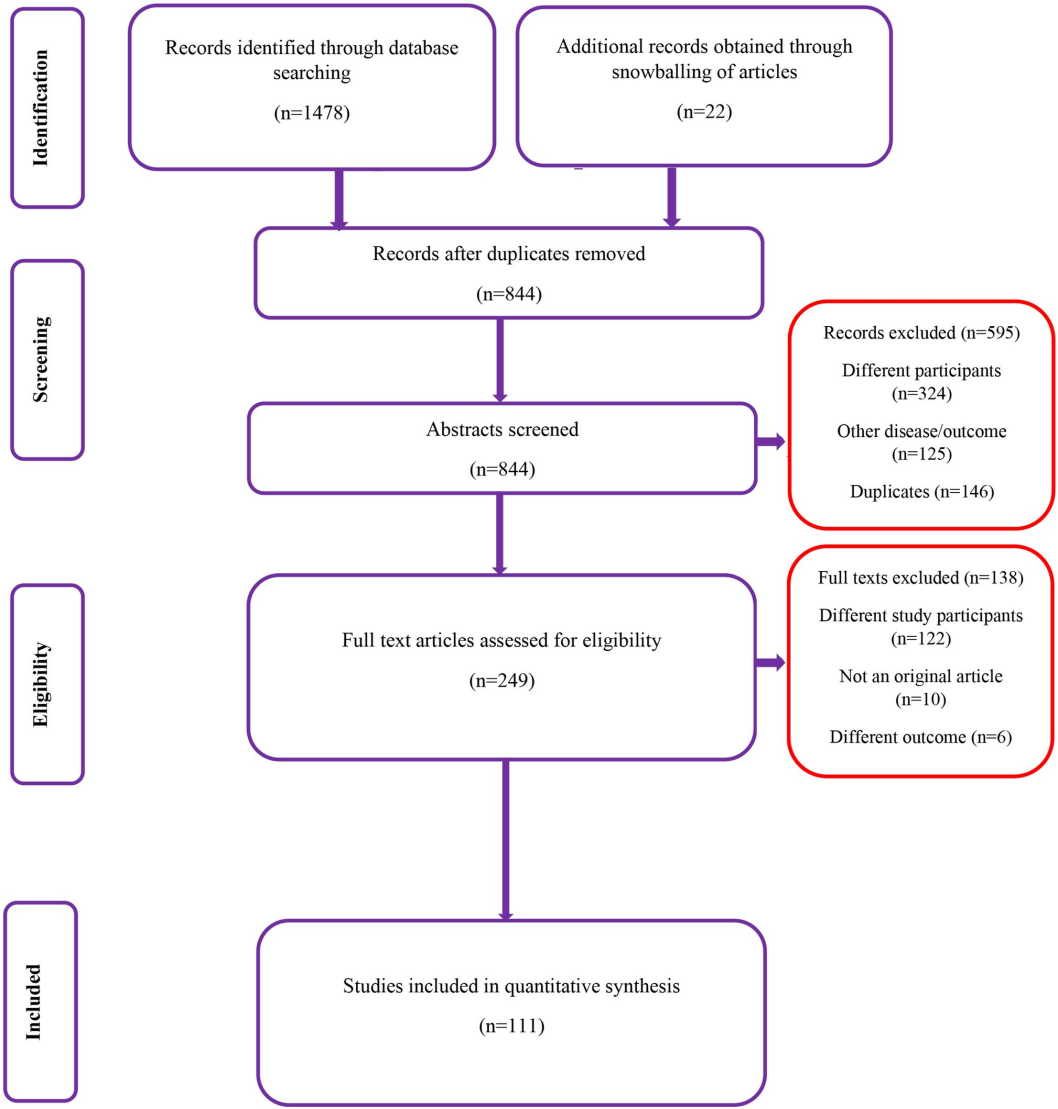
Flow chart showing the search strategy and selection of studies.

We conducted a systematic search to find studies that report the prevalence of MS from the dates of database inception until August 2019. During the first stage of screening (title, abstract and keywords), we retrieved 310 studies from the following databases: Medline, ScienceDirect, Cochrane library and Google Scholar. After removal of duplicates, we reviewed 249 articles’ full texts against our eligibility criteria for the possible inclusion in the review. We reviewed the bibliographies of the retrieved articles and found four more relevant studies. Finally, we analysed 111 studies with 133,926 participants satisfying the inclusion criteria (**Fig 1**) [17–127].

### Characteristics of the included studies

**Table 1** lists the characteristics of the studies analysed. Most of the studies (46) were conducted in Southern states like Andhra Pradesh, Karnataka, Kerala and Tamil Nadu. The mean age of study participants ranged from 19.6 to 69.4 years. Coming to the study setting, 83 were from community-based studies followed by 21 from facility-based studies and 9 from workplace-based studies. The sample sizes in the studies varied from 60 to 9,886. Majority of the studies (49 out of 111) have reported separate estimates for urban region, while 34 studies reported for rural and 4 studies for tribal region. Rest of the studies did not provide separate estimates for urban, rural or tribal regions because of the study setting (facility or workplace-based studies). Majority (82 out of 111) studies have reported estimates for female burden of MS while 76 studies have reported estimates for male adult population.

**Table 1:** Characteristics of the studies included (N = 111).

S.N.	Author and year	State	Geographical Region	Setting	Urban/Rural/Tribal	Age group (years)	Gender	Sample size	Diagnostic criteria	Prevalence (95%CI)
1.	Bal 2011	Chandigarh	North	Facility	NA	25–50	Males & Females	440	NCEP ATP -III	37% (32%-42%)
2.	Bandella 2017	Andhra Pradesh	South	Community	Urban, rural and tribal	20–60	Males & Females	1032	NCEP ATP -III	32% (29%-35%)
3.	Bansal 2009	New Delhi	North	Facility	NA	≥18	Males & Females	1905	NCEP ATP -III	48% (45%-50%)
4.	Bansal 2017	Uttar Pradesh	Central	Facility	NA	30–70	Males & Females	350	NCEP ATP -III	17% (13%-21%)
5.	Bansal A 2015	New Delhi	North	Community	Urban	35–65	Males & Females	406	NCEP ATP -III	75% (71%-79%)
6.	Barik A 2017	West Bengal	East	Community	Rural	≥18	Males & Females	9886	NCEP ATP -III	16% (15%-17%)
7.	Basha 2018	Andhra Pradesh	South	Community	Urban	≥18	Males & Females	802	NCEP ATP -III	32% (29%-35%)
8.	Bhagat 2017	Chandigarh	North	Community	Urban	18–25	Males & Females	611	IDF	18% (15%-22%)
9.	Bhat R 2010	Jammu & Kashmir	North	Facility	NA	≥18	Males & Females	500	NCEP ATP -III	9% (6%-11%)
10.	Bhattacharya 2016	Telangana	South	Facility	NA	≥60	Males & Females	114	NCEP ATP -III	42% (33%-52%)
11.	Bhutia 2017	Sikkim	Northeast	Facility	NA	≥20	Males & Females	361	IDF	34% (29%-39%)
12.	Chakraborty 2015	West Bengal	East	Community	Urban	≥18	Males & Females	690	NCEP ATP -III	33% (29%-36%)
13.	Chhabra 2014	Not mentioned	North	Community	Urban & Rural	25–60	Females	200	NCEP ATP -III	33% (27%-40%)
14.	Chinawale 2018	Gujarat	West	Community	Not mentioned	20–80	Males & Females	473	IDF	41% (37%-46%)
15.	Chow 2007	Andhra Pradesh	South	Community	Rural	≥30	Males & Females	4535	NCEP ATP -III	25% (23%-26%)
16.	Das 2011	West Bengal	East	Community	Urban & Rural	≥30	Males & Females	350	NCEP ATP -III	48% (43%-54%)
17.	Das 2017	West Bengal	East	Community	Not mentioned	18–24	Males & Females	397	IDF	5% (3%-7%)
18.	Das M 2011	West Bengal	East	Community	Urban & Rural	≥30	Males & Females	448	NCEP ATP -III	29% (25%-34%)
19.	Deedwania 2014	11 cities in India	India	Community	Urban	≥20	Males & Females	6198	NCEP ATP -III	36% (35%-37%)
20.	Deepa M 2006	Tamil Nadu	South	Community	Urban	≥20	Males & Females	2350	NCEP ATP -III	18% (17%-20%)
21.	Deshmukh 2012	Maharashtra	Central	Community	Rural	≥18	Males & Females	300	NCEP ATP -III	17% (13%-22%)
22.	Dhabriya 2015	Rajasthan	North	Community	Urban	≥18	Males & Females	1130	IDF	23% (20–25%)
23.	Goyal 2013	Uttarkhand	North	Facility	NA	≥18	Males & Females	380	IDF	21% (17%-26%)
24.	Gupta 2012	Rajasthan	North	Community	Urban	≥20	Males & Females	6106	NCEP ATP -III	26% (25%-27%)
25.	Gupta A 2004	Rajasthan	North	Community	Urban	≥20	Males & Females	1091	NCEP ATP III	32% (29%-34%)
26.	Gupta R 2007	Rajasthan	North	Community	Urban	≥20	Males & Females	644	NCEP ATP III	47% (43%-51%)
27.	Gupta R 2012	Rajasthan	North	Community	Urban	20–60	Males & Females	739	NCEP ATP III	23% (20%-26%)
28.	Harikrishnan 2018	Kerala	South	Community	Urban & Rural	≥20	Males & Females	5063	NCEP ATP III	24% (23%-25%)
29.	Ismail 2016	Kerala	South	Community	Tribal	≥18	Males & Females	120	NCEP ATP III	28% (20%-37%)
30.	Jain 2015	Maharashtra	Central	Facility	NA	18–25	Males & Females	668	NCEP ATP III	11% (9%-14%)
31.	Jeyasheela 2018	Tamil Nadu	South	Facility	NA	≥45	Females	154	IDF	64% (56%-72%)
32.	Jones 2016	Andhra Pradesh	South	Community	Rural	≥18	Males & Females	6217	NCEP ATP III	14% (13%-15%)
33.	Kamble 2010	Maharashtra	Central	Community	Rural	≥18	Males & Females	300	NCEP ATP III	9% (6%-13%)
34.	Kandpal 2016	Uttarkhand	North	Community	Tribal	20–60	Males & Females	288	NCEP ATP III	39% (34%-45%)
35.	Kanjilal 2008	Karnataka, Maharashtra	South & Central	Facility	NA	≥20	Males & Females	2315	NCEP ATP III	58% (56%-60%)
36.	Kapil 2018	Uttarkhand	North	Community	Rural	≥60	Males & Females	979	IDF	29% (26%-32%)
37.	Kaur J 2014	Punjab	North	Community	Urban & Rural	≥20	Males & Females	351	NCEP ATP III	17% (14%-22%)
38.	Kaur P 2010	Tamil Nadu	South	Workplace	NA	≥20	Males	1077	IDF	41% (38%-44%)
39.	Kaushal 2016	Uttar Pradesh	Central	Community	Urban	≥20	Males & Females	127	NCEP ATP III	37% (29%-46%)
40.	Kempegowda 2011	Karnataka	South	Community	Rural	≥20	Males & Females	495	NCEP ATP III	20% (16%-23%)
41.	Khan 2018	Uttar Pradesh	Central	Facility	NA	≥20	Males & Females	420	NCEP ATP III	41% (36%-46%)
42.	Kotokey 2013	Assam	Northeast	Community	Urban	≥20	Males & Females	502	IDF	33% (29%-37%)
43.	Kunti 2019	West Bengal	East	Facility	NA	≥18	Males & Females	330	NCEP ATP III	64% (58%-69%)
44.	Lakshmipriya 2012	Tamil Nadu	South	Community	Urban	≥20	Males & Females	1875	IDF	28% (26%-30%)
45.	Lateef 2007	Andhra Pradesh	South	Community	Urban	≥20	Males & Females	1519	NCEP ATP III	24% (22%-27%)
46.	Lavanya 2012	Andhra Pradesh	South	Community	Urban	≥20	Males & Females	350	NCEP ATP III	23% (19%-28%)
47.	Madan G 2016	Maharashtra	West	Community	Urban & Rural	18–65	Males	313	IDF	40% (34%-46%)
48.	Madhav 2013	Andhra Pradesh	South	Workplace	NA	≥20	Males	180	IDF	22% (16%-28%)
49.	Mahadik 2007	Maharashtra	West	Community	Urban & Rural	≥20	Males & Females	1071	NCEP ATP III	24% (22%-27%)
50.	Mahajan 2012	New Delhi	North	Community	Urban & Rural	≥20	Males & Females	7174	NCEP ATP III	57% (56%-58%)
51.	Mahanta 2013	Assam	Northeast	Community	Urban & Rural	≥35	Males & Females	297	NCEP ATP III	27% (22%-32%)
52.	Mahanta 2017	Assam	Northeast	Community	Urban & Rural	20–60	Males & Females	3372	NCEP ATP III	48% (46%-49%)
53.	Majumdar 2011	Karnataka	South	Community	Not available	18–75	Males & Females	441	NCEP ATP III	28% (24%-32%)
54.	Majumdar 2017	Andhra Pradesh	South	Community	Urban	≥60	Males & Females	112	IDF	54% (45%-64%)
55.	Mangat 2010	Chandigarh	North	Community	Urban & Rural	≥18	Males & Females	605	NCEP ATP III	39% (35%-43%)
56.	Manjunath 2014	Andhra Pradesh	South	Community	Urban	18–25	Males & Females	473	NCEP ATP III	4% (2%-6%)
57.	Mini 2018	Kerala	South	Workplace	NA	18–64	Males & Females	2287	NCEP ATP III	19% (18%-21%)
58.	Misra 2011	Haryana	North	Community	Rural	≥20	Males & Females	307	NCEP ATP III	12% (9%-16%)
59.	Mittal 2018	Uttar Pradesh	Central	Community	Urban & Rural	≥20	Males & Females	125	IDF	35% (27%-44%)
60.	Mohan 2007	Tamil Nadu	South	Community	Urban & Rural	≥20	Males & Females	1736	NCEP ATP III	17% (15%-19%)
61.	Mohan 2009	Tamil Nadu	South	Community	Urban & Rural	≥20	Males & Females	541	NCEP ATP III	37% (33%-41%)
62.	Nag 2015	West Bengal	East	Community	Rural	≥20	Males & Females	1007	IDF	26% (23%-29%)
63.	Naik 2016	Andhra Pradesh	South	Community	Urban	≥60	Males & Females	295	IDF	35% (29%-40%)
64.	Nithya 2015	Tamil Nadu	South	Community	Rural	≥60	Males & Females	514	NCEP ATP III	20% (17%-24%)
65.	Pai 2019	Karnataka	South	Community	Urban & Rural	≥20	Males & Females	420	NCEP ATP III	4% (2%-6%)
66.	Pandey 2010	Maharashtra	West	Community	Urban	35–65	Females	498	IDF	57% (52%-61%)
67.	Parale 2008	Karnataka	South	Workplace	NA	≥30	Males & Females	700	NCEP ATP III	27% (24%-30%)
68.	Patel 2016	Gujarat	West	Facility	NA	25–65	Males & Females	478	NCEP ATP III	26% (22%-30%)
69.	Pathak 2018	Haryana	North	Community	Rural	≥20	Males & Females	1200	IDF	9% (8%-11%)
70.	Pemminati 2010	Karnataka	South	Community	Urban	≥20	Males & Females	451	IDF	30% (26%-34%)
71.	Prabhakaran 2005	New Delhi	North	Workplace	NA	20–59	Males	2120	NCEP ATP III	35% (33%-37%)
72.	Prabhakaran 2007	New Delhi	North	Community	Urban & Rural	35–64	Males & Females	4044	NCEP ATP III	33% (32%-35%)
73.	Pradeepa 2016	Tamil Nadu	South	Community	Urban	≥60	Males & Females	222	IDF	37% (31%-44%)
74.	Prakash 2013	Uttar Pradesh	North	Facility	NA	≥18	Males & Females	1340	NCEP ATP III	32% (30%-35%)
75.	Prasad 2012	Orissa	East	Community	Urban	20–80	Males & Females	1178	IDF	43% (40%-46%)
76.	Rajput 2014	Haryana	North	Community	Urban & Rural	≥20	Males & Females	3042	IDF	29% (27%-31%)
77.	Ramachandran 2003	Tamil Nadu	South	Community	Not mentioned	20–75	Males & Females	475	NCEP ATP III	41% (37%-46%)
78.	Ramachandran 2008	Tamil Nadu	South	Workplace	NA	≥20	Males & Females	2499	NCEP ATP III	29% (27%-31%)
	Ramachandran 2008	Tamil Nadu	South	Community	Urban	≥20	Males & Females	3278	NCEP ATP III	41% (37%-46%)
79.	Randhwa 2015	Punjab	North	Community	Rural	25–55	Females	300	NCEP ATP III	26% (21%-31%)
80.	Ravikiran 2010	Chandigarh	North	Community	Urban & Rural	≥20	Males & Females	2225	NCEP ATP III	45% (43%-47%)
81.	Roopa 2010	Tamil Nadu	South	Community	Urban	≥20	Males & Females	358	NCEP ATP III	35% (30%-40%)
82.	Sachdev 2009	New Delhi	North	Community	Urban	26–32	Males & Females	1492	IDF	25% (23%-27%)
83.	Sarkar P 2016	Karnataka	South	Community	Rural	≥30	Males & Females	448	NCEP ATP III	26% (22%-30%)
84.	Sarkar S 2006	West Bengal	East	Community	Tribal	≥20	Males & Females	588	NCEP ATP III	25% (22%-29%)
85.	Sarma 2015	Andhra Pradesh	South	Community	Rural	30–50	Females	60	NCEP ATP III	20% (11%-32%)
86.	Sawant 2011	Maharashtra	West	Community	Urban	≥20	Males & Females	548	NCEP ATP III	20% (16%-23%)
87.	Selvaraj 2012	Tamil Nadu	South	Community	Rural	30–50	Females	150	NCEP ATP III	31% (23%-39%)
88.	Selvaraj 2019	Tamil Nadu	South	Community	Rural	20–40	Males	360	NCEP ATP III	17% (13%-21%)
89.	Shalini 2013	Karnataka	South	Community	Urban & Rural	≥18	Females	1023	NCEP ATP III	57% (54%-60%)
90.	Sharma MK 2018	Uttar Pradesh	Central	Community	Urban & Rural	20–55	Males & Females	290	NCEP ATP III	20% (16%-25%)
91.	Sharma R 2019	Jammu & Kashmir	North	Community	Not mentioned	≥18	Males & Females	210	NCEP ATP III	35% (29%-42%)
92.	Sharma S 2016	Chandigarh	North	Facility	NA	45–55	Females	350	NCEP ATP III	63% (57%-68%)
93.	Sharma S 2016	Karnataka	South	Workplace	NA	20–50	Males & Females	210	NCEP ATP III	12% (8%-17%)
94.	Singh 2016	Haryana	North	Community	Rural	≥20	Males & Females	1700	IDF	27% (25%-29%)
95.	Singh 2017	Uttar Pradesh	North	Community	Urban	≥25	Males & Females	2002	NCEP ATP III	19% (18%-21%)
96.	Sinha N 2016	Telangana	South	Community	Urban	≥60	Males & Females	114	IDF	42% (33%-52%)
97.	Sinha S 2012	New Delhi	North	Community	Urban	≥20	Females	226	NCEP ATP III	30% (24%-36%)
98.	Srimani 2017	West Bengal	East	Community	Rural	45–70	Females	222	IDF	46% (39%-53%)
99.	Srimani 2018	West Bengal	East	Community	Rural	45–70	Females	509	IDF	38% (34%-42%)
100.	Srinivasan 2016	Kerala	South	Facility	NA	≥20	Males & Females	432	NCEP ATP III	61% (56%-66%)
101.	Subramani 2018	Madhya Pradesh	Central	Facility	NA	20–79	Males & Females	1190	NCEP ATP III	50% (47%-53%)
102.	Tandon 2010	Jammu & Kashmir	North	Community	Rural	≥45	Females	500	NCEP ATP III	13% (10%-16%)
103.	Tharkar 2010	Tamil Nadu	South	Community	Urban	≥20	Males & Females	2021	NCEP ATP III	32% (30%-34%)
104.	Tharkar S 2008	Tamil Nadu	South	Workplace	NA	≥30	Males & Females	318	IDF	57% (52%-63%)
	Tharkar S 2008	Tamil Nadu	South	Community	Urban	≥30	Males & Females	410	IDF	28% (23%-32%)
105.	Thayyil 2012	Kerala	South	Workplace	NA	≥30	Males	823	NCEP ATP III	17% (14%-19%)
106.	Thiruvagounder 2010	Tamil Nadu	South	Facility	NA	≥20	Males & Females	1568	NCEP ATP III	29% (27%-31%)
107.	Thyagi 2013	Uttar Pradesh	Central	Facility	NA	25–65	Males & Females	405	NCEP ATP III	43% (38%-48%)
108.	Vatakanchery 2019	Kerala	South	Facility	NA	20–60	Males & Females	520	IDF	76% (72%-80%)
109.	Venugopal 2019	Pondicherry	South	Community	Rural	≥30	Males & Females	489	IDF	40% (35%-44%)
110.	Wani 2014	Jammu & Kashmir	North	Facility	NA	20–60	Males & Females	500	NCEP ATP III	9% (6%-11%)
111.	Zafar 2017	Uttar Pradesh	West	Community	Rural	18–55	Males & Females	2982	NCEP ATP III	12% (11%-13%)

NCEP ATP III–National Cholesterol Education Program Adult Treatment Panel III.

IDF–International Diabetes Federation.

NA–Not applicable.

### Methodological quality of the included studies

We performed assessments of risk of bias for all the included studies using NO scale. First, selection bias domains were assessed and it was found that 64 out of 111 studies (58% of studies) had high risk of bias related to representativeness of the sample for our review, 80 (72%) studies did not report proper justification for sample size, 69 (62%) studies did not report non-response rate or their characteristics. All the studies reported use of validated measurement tool. Under the outcome domain, only one study did not independently assess the outcome; nine studies did not report the statistical methods used in the study. Almost one-third i.e. 35 (31.5%) of the included studies had high risk of bias as per NO scale.

### Burden of metabolic syndrome in India

**Fig 2:**
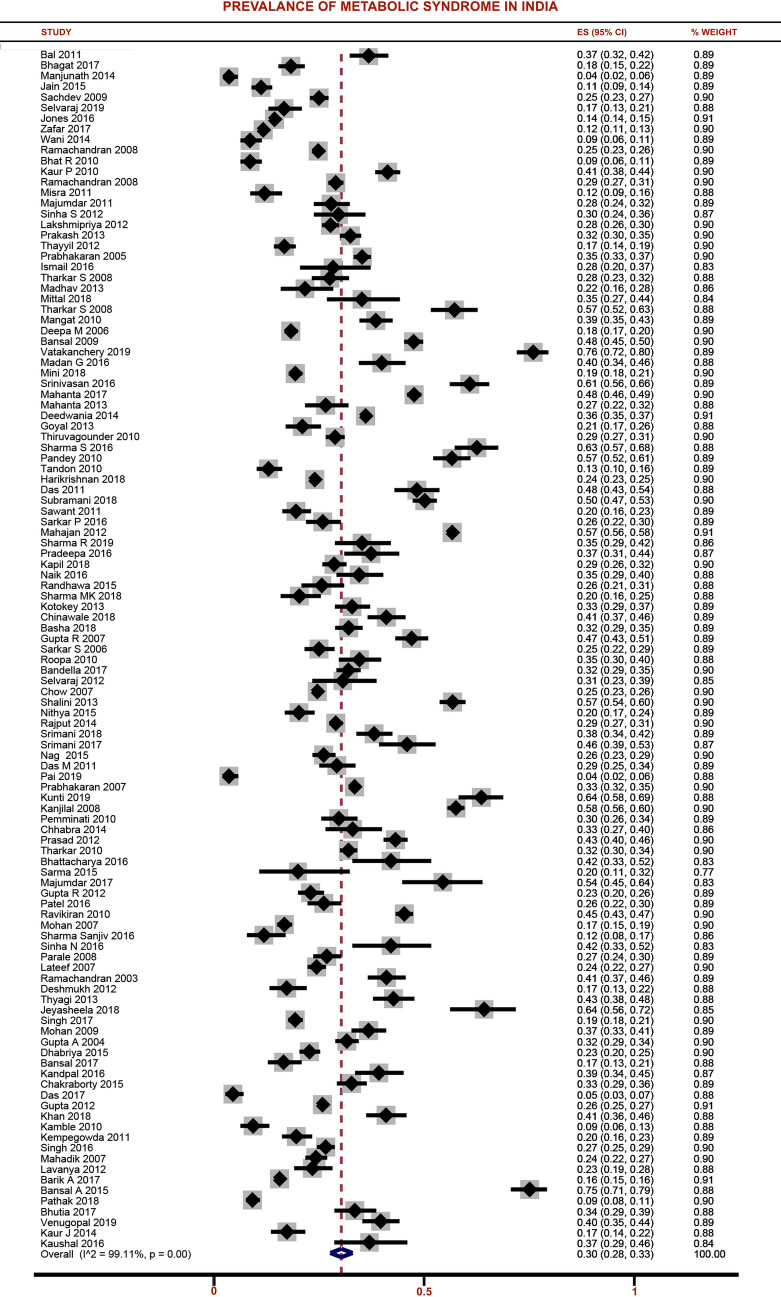
Forest plot showing the prevalence of metabolic syndrome among adult population in India.

Pooled estimate was calculated after adjusting for population size weights and graphically depicted in **Fig 2**. The overall pooled prevalence of MS among adult population in India was 30% (95%CI: 28%-33%).

State wise analysis of MS showed that the maximum prevalence of MS was reported in Madhya Pradesh (50%) followed by New Delhi (43%), Odisha (43%) and Telangana (42%). Least pooled prevalence of MS was found in Jammu & Kashmir (15%) followed by Haryana (18%) and Punjab (21%). There was a significant heterogeneity found among the studies included in our review (I^2^ = 99.1%; p<0.001). Additional subgroup analysis was performed to explore the source of heterogeneity and obtain separate estimates based on age group, gender, study setting, geographical area and regions, year of publication, representativeness of sample, waist circumference measurement, quality of studies and diagnostic criteria **(Table 2)**.

**Table 2:** Summary of findings and subgroup analysis of studies reporting prevalence of metabolic syndrome in India.

*Characteristic*	*Number of studies pooled*	*Pooled ES*^*#*^ *(95% CI)*	*I*^*2*^	*P for heterogeneity/trend*
Pooled prevalence of Metabolic syndrome in India = 30% (95%CI: 28%-33%)				
**AGE GROUP**				
18–29 years	12	13% (8%-18%)	93.3%	**<0.001**
30–39 years	11	32% (24%-41%)	96.7%	
40–49 years	11	41% (33%-48%)	95.9%	
50–59 years	10	50% (43%-57%)	94.3%	
≥60 years	17	41% (34%-49%)	97.3%	
**GENDER**				
Male	76	26% (22%-29%)	98.6%	**0.04**
Female	82	35% (31%-38%)	98.8%	
**URBAN VS RURAL VS TRIBAL**				
Urban	49	32% (29%-36%)	98.9%	**<0.001**
Rural	34	22% (20%-25%)	97.7%	
Tribal	4	28% (21%-36%)	88.4%	
**STUDY SETTING**				
Community	83	29% (26%-32%)	99.1%	0.08
Facility	21	38% (30%-47%)	99.1%	
Workplace	9	28% (22%-35%)	98.2%	
**GEOGRAPHICAL REGION**				
North	31	30% (25%-35%)	99.2%	0.83
South	46	30% (26%-33%)	98.5%	
East	11	33% (23%-43%)	99.1%	
West	7	30% (19%-44%)	99%	
Central	10	27% (17%-39%)	98.4%	
Northeast	4	35% (25%-46%)	96.7%	
**DIAGNOSTIC CRITERIA**				
NTEP-ATP III	81	29% (26%-32%)	99.2%	**0.05**
IDF	32	34% (30%-39%)	98.3%	
**WAIST CIRCUMFERENCE MEASUREMENT**				
Tip of iliac crest	34	29% (25%-33%)	98.6%	0.67
Midline of iliac crest & costal margin	22	32% (26%-39%)	99%	
**YEAR OF PUBLICATION**				
2003–2006	5	30% (22%-39%)	98.2%	0.62
2007–2010	26	32% (27%-36%)	98.9%	
2011–2014	30	27% (23%-33%)	99.1%	
2015–2019	52	31% (27%-36%)	99.1%	
**SAMPLING (REPRESENTATIVENESS)**				
Random	47	29% (25%-33%)	99.3	0.37
Non-random	64	31% (27%-35%)	98.8%	
**QUALITY OF STUDIES**				
Low	35	32% (28%-36%)	98.4%	0.40
High	76	29% (26%-33%)	99.2%	

#### Age wise distribution of metabolic syndrome in India

The pooled prevalence of MS differed significantly across the age groups (p<0.001). There was a steady increase in the burden across the age groups from 13% (18–29 years group) to 50% (50–59 years). There was significant heterogeneity among the studies reporting prevalence across all the age groups with I^2^>90% and p<0.001.

#### Gender distribution of metabolic syndrome in India

In total, 82 studies have reported prevalence of MS among female adult population while 76 studies have reported for males. The pooled prevalence of MS among adult females in India was 35% (95%CI: 31%-38%); while for males, the pooled prevalence was 26% (95%CI: 22%-29%). There was significant heterogeneity among the studies reporting prevalence in females and males (I^2^ = 98%, p<0.001).

#### Urban vs rural vs tribal

Overall, 49 studies have reported separate estimates for urban region, 34 for rural and 4 for tribal region. The pooled prevalence of MS among adult urban population in India was 32% (95%CI: 29%-36%); while in rural population, the pooled prevalence was 22% (95%CI: 20%-25%) and in tribal population, it was 28% (95%CI: 21%-36%). Significant heterogeneity was found among the studies reporting prevalence across urban, rural and tribal regions (p<0.001).

#### Study setting (community vs facility vs workplace)

Subgroup analysis based on study setting showed that the facility-based studies showed higher prevalence (38%; 95%CI: 30%-47%) followed by community based (29%; 95%CI: 26%-32%) and workplace-based studies (28% 95%CI: 22%-35%). However, this difference was not statistically significant (p = 0.08). There was significant heterogeneity among the studies reporting prevalence of MS irrespective of the study setting (p<0.001).

#### Geographical regions

*North India*. In total, 31 studies reporting the burden of MS were from Northern region. The pooled prevalence of MS in North India was 30% (95%CI: 25%-35%). There was a significant heterogeneity found among the studies conducted in North India (I^2^ = 99.2%; p<0.001). Among the 31 studies, 15 reported separate estimates for urban and 10 for rural regions. Prevalence of MS in urban North India was 35% (95%CI: 27%-44%; I^2^ = 99.3%; p<0.001) while for rural North India, the pooled prevalence was 21% (95%CI: 15%-27%; I^2^ = 97.4%, p<0.001). We also checked for gender wise distribution of MS in North India. The prevalence of MS among adult females in North India was 33% (95%CI: 26%-40%; I^2^ = 98.9%, p<0.001); while for adult male population, it was 26% (95%CI: 20%-34%; I^2^ = 99%, p<0.001) **(S1 Fig)**.

*Central India*. Ten studies have reported prevalence of MS in Central India. The pooled estimate for prevalence of MS in Central India was 27% (95%CI: 17%-39%; I^2^ = 98.4%, p<0.001). Only one study was conducted in urban community of Central India. It reported a prevalence of 25% with 95%CI: 18%-33%. Three studies were conducted in the rural community of Central India, with which we found a pooled prevalence of 14% with 95%CI: 9%-20%. Gender wise distribution showed that the prevalence of MS was higher among adult females in Central India (30%; 95%CI: 17%-46%; I^2^ = 97.3%, p<0.001) when compared to males (21%; 95%CI: 12%-33%; I^2^ = 97.2%, p<0.001) (**S2 Fig).**

*Western India*. In total, 7 studies from Western India were included in our review. The pooled prevalence of MS in Western India was 30% (95%CI: 19%-44%; I^2^ = 99%, p<0.001). There was a significant urban-rural difference in the prevalence of MS in Western India. The prevalence of MS in urban Western India was 37% (95%CI: 21%-55%, I^2^ = 98.1%, p<0.001); while in rural Western India, it was 13% (95%CI: 12%-15%). Gender wise distribution of MS showed that the prevalence was higher among females (32%; 95%CI: 15%-51%; I^2^ = 98.3%, p<0.001) when compared to males (25%; 95%CI: 14%-35%; I^2^ = 95%, p<0.001) **(S3 Fig)**.

*Eastern India*. Eleven studies have reported the prevalence of MS in Eastern India. The pooled prevalence of MS in Eastern India was 33% (95%CI: 23%-43%; I^2^ = 99.1%, p<0.001). The prevalence was higher among urban Eastern India (38%; 95%CI: 31%-45%; I^2^ = 90.3%, p<0.001) when compared to rural Eastern India (29%; 95%CI: 18%-40%; I^2^ = 98.5%, p<0.001). Gender distribution of MS showed that the burden was higher among the females (39%; 95%CI: 27%-53%; I^2^ = 98.8%, p<0.001) when compared to males (23%; 95%CI: 12%-36%, I^2^ = 98.5%, p<0.001) **(S4 Fig)**.

*Southern India*. In total, 46 studies reporting the burden of MS were from Southern region. The pooled prevalence of MS in South India was 30% (95%CI: 26%-33%). There was a significant heterogeneity found among the studies conducted in South India (I^2^ = 98.6%; p<0.001). Prevalence of MS in urban South India was 30% (95%CI: 25%-34%; I^2^ = 98.1%; p<0.001) while for rural South India, the pooled prevalence was 25% (95%CI: 20%-30%; I^2^ = 97.9%, p<0.001). Gender wise prevalence of MS in South India was also checked. The prevalence of MS among adult females in South India was 34% (95%CI: 28%-40%; I^2^ = 98.1%, p<0.001); while for adult male population, it was 26% (95%CI: 21%-31%; I^2^ = 97.9%, p<0.001) **(S5 Fig)**.

*Northeast India*. Only 4 studies were conducted in Northeast India to determine the prevalence of MS. The pooled prevalence of MS in Northeast India was 35% (95%CI: 25%-46%). There was a significant heterogeneity found among the studies conducted in South India (I^2^ = 96.9%; p<0.001) **(S6 Fig)**. There was no sufficient number of studies to see the urban-rural and gender-based differences in the burden of MS.

### Additional subgroup analysis and sensitivity analysis

Subgroup analysis based on diagnostic criteria revealed a statistically significant difference in the pooled estimate between NCEP ATP-III and IDF criteria (29% vs 34%; p = 0.05). However, we did not see any statistically significant difference in the burden of MS based on year of publication, difference in measurement of waist circumference, quality of studies or representativeness of sample.

We performed sensitivity analysis by excluding the studies conducted exclusively on elderly (≥60 years), studies having high risk of bias or not conducted on a representative sample, and studies conducted before 2007. This is to provide robust and latest estimate on the burden of MS among adult population in India. In total, 47 out of the 111 studies satisfied these criteria and included in the sensitivity analysis. The pooled prevalence of MS obtained in sensitivity analysis was still almost similar to the overall findings (29%; 95%CI: 25%-33%) indicating that the estimate is robust to the changes in age group, year, and quality of studies.

### Meta-regression

First, we conducted univariate meta-regression with individual study level factors such as study setting, geographical region, diagnostic criteria, year of publication, mean age, representativeness of the sample and quality of studies **(Table 3)**. We found significant association with mean age and study setting. However, we performed multivariable meta-regression with factors having p value less than 0.2 such as study setting, mean age, and diagnostic criteria used. The adjusted model was able to explain 24.5% of the between-study variability and the model was statistically significant (p = 0.004).

**Table 3:** Univariate and multivariate meta-regression performed to explore the heterogeneity between the studies (N = 113).

Characteristics	Unadjusted Co-efficient	Unadjusted p-Value	Adjusted Co-efficient	Adjusted p-value
**Mean age**	0.0069	**0.002**	0.0068	**0.002**
**Study setting**				
Community	Ref	-	Ref	-
Facility	0.10	**0.006**	0.10	**0.04**
Workplace	-0.004	0.93	0.04	0.49
**Diagnostic criteria**				
IDF	Ref	**-**	Ref	-
NCEP-ATP	-0.05	0.10	-0.07	0.11
**Year of publication**			{Not included in the multivariate model}	
2003–2006	Ref	-		
2007–2010	0.02	0.75		
2011–2014	-0.01	0.82		
2015–2019	0.02	0.75		
**Geographical region**				
North	Ref	-		
South	0.05	0.41		
East	0.02	0.61		
West	0.07	0.40		
Central	0.02	0.63		
Northeast	0.03	0.69		
**Representativeness of the sample**				
Representative	Ref	-		
Non-representative	0.03	0.34		
**Quality of studies**				
Low risk	Ref	-		
High risk	-0.02	0.46		

### Publication bias

**Fig 3:**
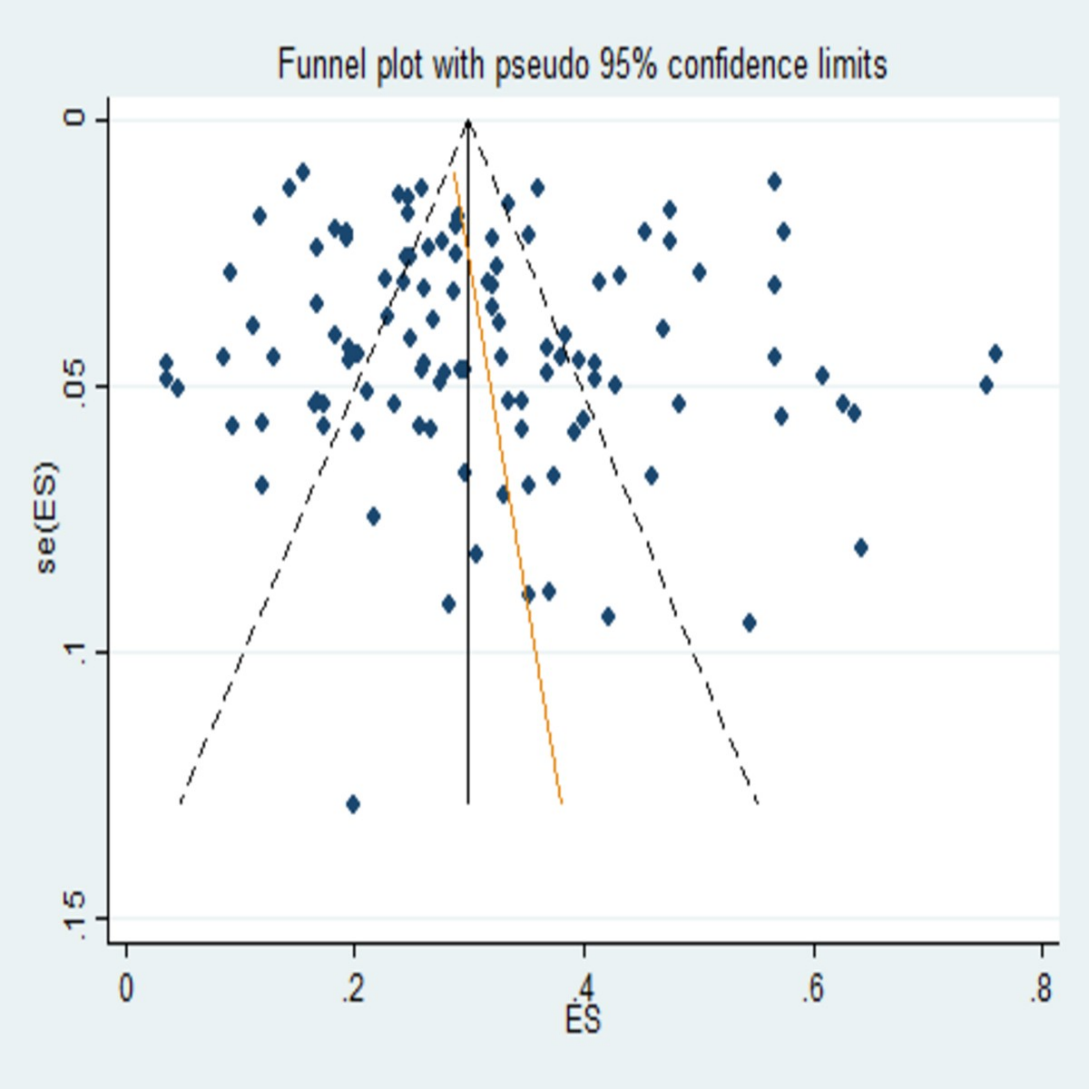
Funnel plot checking the publication bias in the current review.

Egger’s test was performed for the assessment of publication bias. There were no small study effects with non-significant coefficient value (Co-efficient: 0.78; 95%CI: -0.95 to 2.52; p = 0.372) which shows lack of evidence of publication bias. Graphical representation of the test of publication bias was depicted through funnel plot in **Fig 3**. Funnel plot also showed symmetric plot indicating the absence of publication bias.

## Discussion

We have conducted this review to obtain a comprehensive estimate of burden of MS among adult population in India. We have also captured the gender distribution, urban-rural-tribal differences and geographical region wise estimates to find any significant difference in the estimates of MS. In total, we analysed data from 111 studies with 133,926 participants. Majority of the studies were conducted in Southern states followed by Northern states. Majority (83) were from community-based studies. Out of these, 49 have reported separate estimates for urban region, 34 for rural and 4 for tribal region. Majority of the included studies (76 out of 111) had low risk of bias.

The prevalence of MS among adult population in India was 30% (95%CI: 28%-33%). Similar reviews published from other low middle income countries in Middle East, South East Asia and Latin American region also reported that almost one third of general population have MS [128–130]. This recent upsurge in the prevalence of MS among low middle income countries including India might be directly linked with rapid economic development and urbanization in the country. This rapid industrialization can influence drastic changes in lifestyle patterns and nutrition [131–133].

We also found that people living in urban areas (32%; 95%CI: 29%-36%) were found to have higher prevalence of MS when compared to people living in tribal (28%; 95%CI: 21%-36%) or rural areas (22%; 95%CI: 20%-25%). These findings were in line with the studies conducted in neighbouring low middle-income countries [129]. Unhealthy lifestyles, better socio-economic status, decreased physical activity, stress, excessive salt and red meat consumption might be the contributory factors influencing the higher prevalence of MS in urban areas [134].

Age wise distribution showed that there is a steady rise in the burden of MS with increase in age groups and it differed significantly (p<0.001). Gender distribution of MS showed that the females had higher prevalence (35%; 95%CI: 31%-38%) when compared to males 26% (95%CI: 22%-29%). Similar reviews in Eastern Mediterranean [135], South East Asian [136], and Western pacific [137] regions abide by our findings. Major reason for this finding could be the gender specific risk factors such as menopause, polycystic ovarian syndrome and use of hormonal contraceptives among women [138]. Other probable reasons could be the excess risk women carries in terms of elevated body weight, increased waist girth, and low high density lipoprotein cholesterol when compared to men [139–142].

Highest prevalence of MS was reported in Madhya Pradesh (50%) followed by New Delhi (43%), Orissa (43%) and Telangana (42%). Stratified analysis across the geographical regions showed that people living in Northeast India (35%) have highest prevalence of MS followed by Eastern India (33%), regions with unique lifestyle and culture [142]. This highlights the need of understanding the influence of sociocultural, ethno-geographical factors in determining the risk of MS. This high prevalence among the north eastern states could also be due to lesser number of studies (n = 4) from the region. Hence, the current review may not be representative of the entire north eastern population. Further studies should be carried out in this region to have conclusive evidence on the burden of MS.

The major strength of the study is that we have tried to provide the first comprehensive review on burden of MS among adult general population in India. We also provided estimates based on gender, study setting, community and geographical regions. Test for publication bias have found that there was no significant bias in the current review. However, our review has certain limitations. Summarizing and concluding the burden of MS as 30% among adults in India with demographic and socio-economic differences is difficult because of the inherent heterogeneity. We have tried to overcome this limitation by conducting subgroup analysis based on study setting, geographical regions and provided individual prevalence estimates. The chi square test for heterogeneity also revealed significant variability across the national and region wise included studies. Hence, we tried to explain the between-study variability using meta-regression and found the potential sources of heterogeneity.

In spite of these limitations, current review provides important baseline information on the burden of MS among adult population in India. Our review has showed a higher prevalence among women, people living in urban areas and in specific geographical pockets. The findings of our review highlight that MS is a major public health problem in India. It is necessary for the government to allocate adequate resources and establish appropriate cost-effective interventions to tackle the burden of MS. Development and implementation of policies and protocols for the screening (focusing on population level screening strategies) would enable us in early diagnosis and treatment. Special focus should be given towards the vulnerable and high-risk groups. A wholesome comprehensive health care approach encompassing various levels of health care is necessary to achieve better management of MS.

This crucial step would not only enable us to reduce the mortality imposed by its individual components but also to curtail the out-of-pocket expenditure incurred by these conditions. Further comprehensive meta-analysis on population-based studies is necessary to find the factors responsible for MS which will help the policy makers, especially in the low and low middle-income regions to devise region specific interventions.

## Supporting information

S1 Checklist PRISMA 2009 checklist. (DOC)

S1 Fig Forest plot showing the setting wise and gender wise distribution of metabolic syndrome in North India a) North India–Male b) North India–Female c) North India–Urban d) North India–Rural.
(TIFF)

S2 Fig Forest plot showing the setting wise and gender wise distribution of metabolic syndrome in Central India a) Central India–Male b) Central India–Female c) Central India–Rural.
(TIFF)

S3 Fig Forest plot showing the setting wise and gender wise distribution of metabolic syndrome in Western India a) Western India–Male b) Western India–Female c) Western India–Urban d) Western India–Rural.
(TIFF)

S4 Fig Forest plot showing the setting wise and gender wise distribution of metabolic syndrome in Eastern India a) Eastern India–Male b) Eastern India–Female c) Eastern India–Urban d) Eastern India–Rural.
(TIFF)

S5 Fig Forest plot showing the setting wise and gender wise distribution of metabolic syndrome in South India a) South India–Male b) South India–Female c) South India–Urban d) South India–Rural.
(TIFF)

S6 Fig Forest plot showing the setting wise and gender wise distribution of metabolic syndrome in Northeast India a) Northeast India–Male b) Northeast India–Female.
(TIFF)

S1 File Search strategy. (PDF)

S2 File (DTA)
