# Supplementary figures and images for: Prevalence of metabolic syndrome among adult population in India: A systematic review and meta-analysis
Source: PLoS One. 2020 Oct 19;15(10):e0240971. doi: 10.1371/journal.pone.0240971 (PMC7571716; doi:10.1371/journal.pone.0240971)

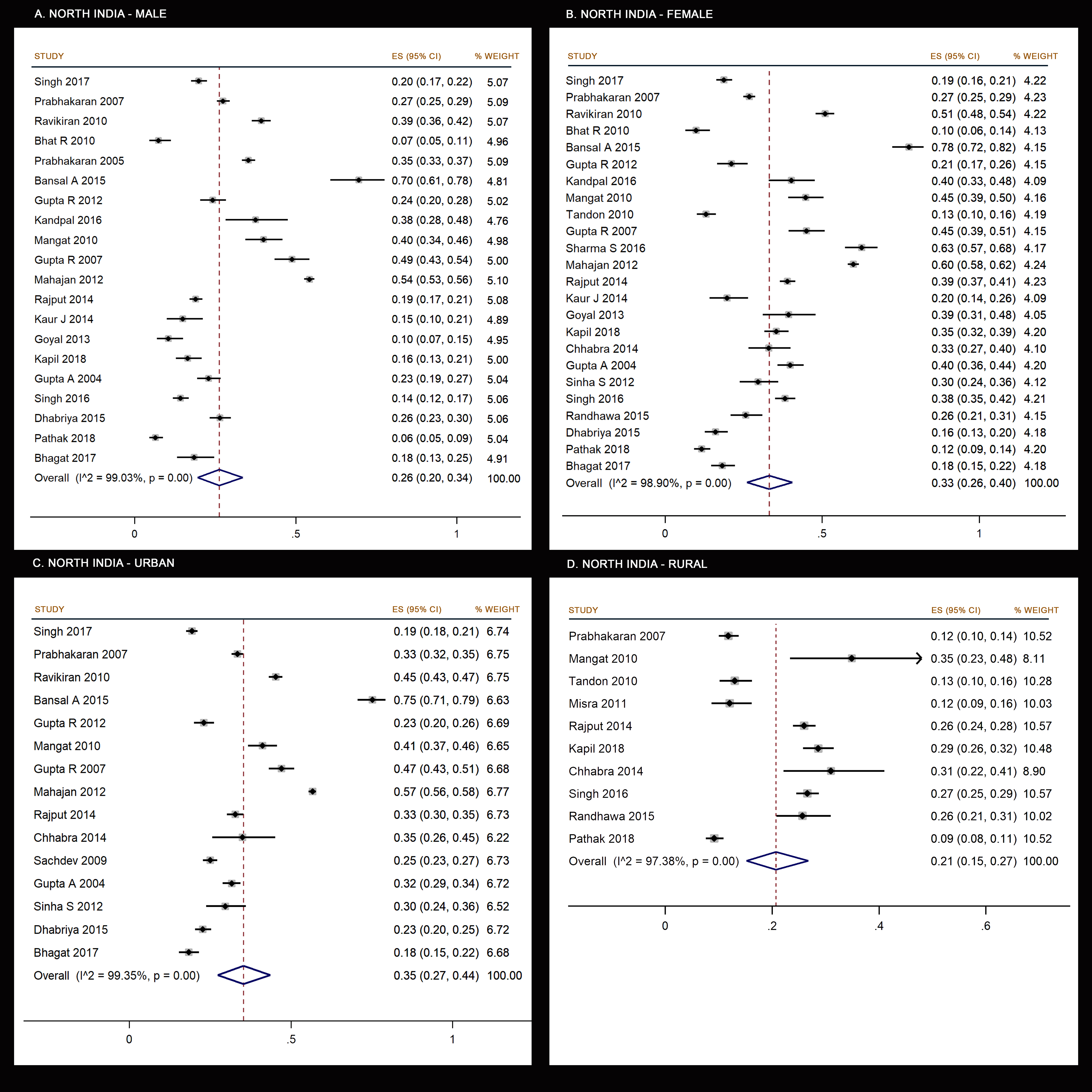

Supplement: S1 Fig — Forest plot showing the setting wise and gender wise distribution of metabolic syndrome in North India a) North India–Male b) North India–Female c) North India–Urban d) North India–Rural. (TIFF) [file pone.0240971.s002.tiff]

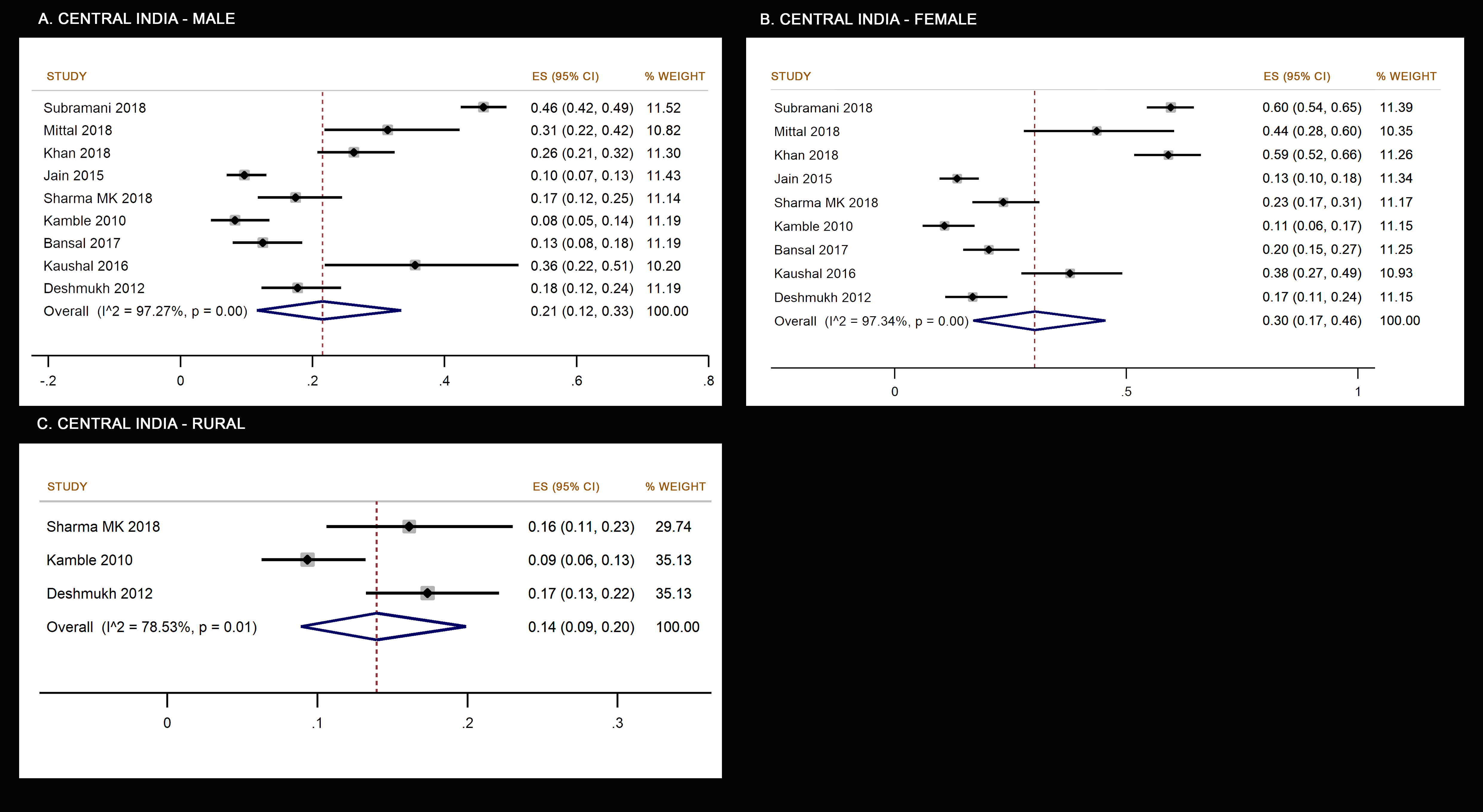

Supplement: S2 Fig — Forest plot showing the setting wise and gender wise distribution of metabolic syndrome in Central India a) Central India–Male b) Central India–Female c) Central India–Rural. (TIFF) [file pone.0240971.s003.tiff]

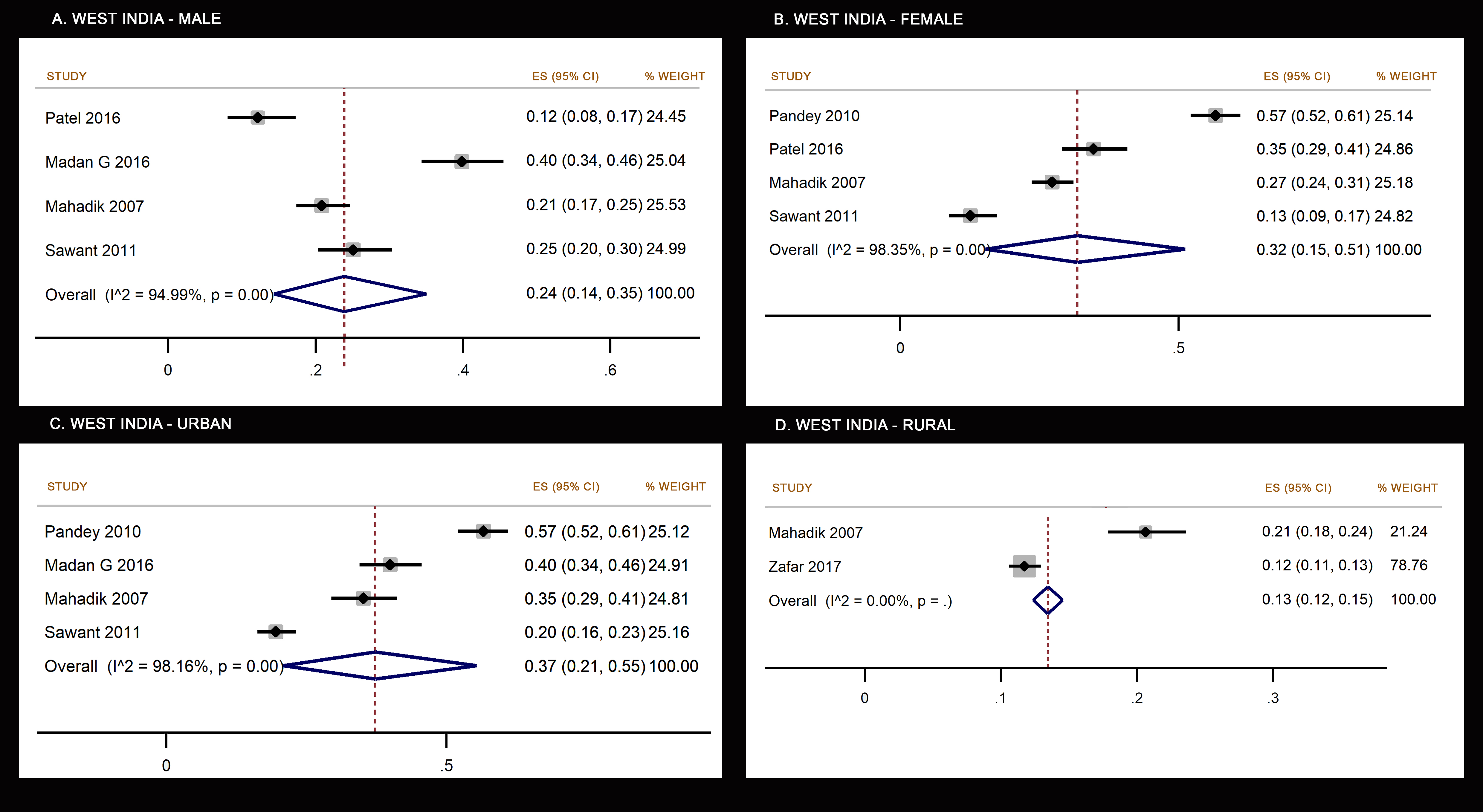

Supplement: S3 Fig — Forest plot showing the setting wise and gender wise distribution of metabolic syndrome in Western India a) Western India–Male b) Western India–Female c) Western India–Urban d) Western India–Rural. (TIFF) [file pone.0240971.s004.tiff]

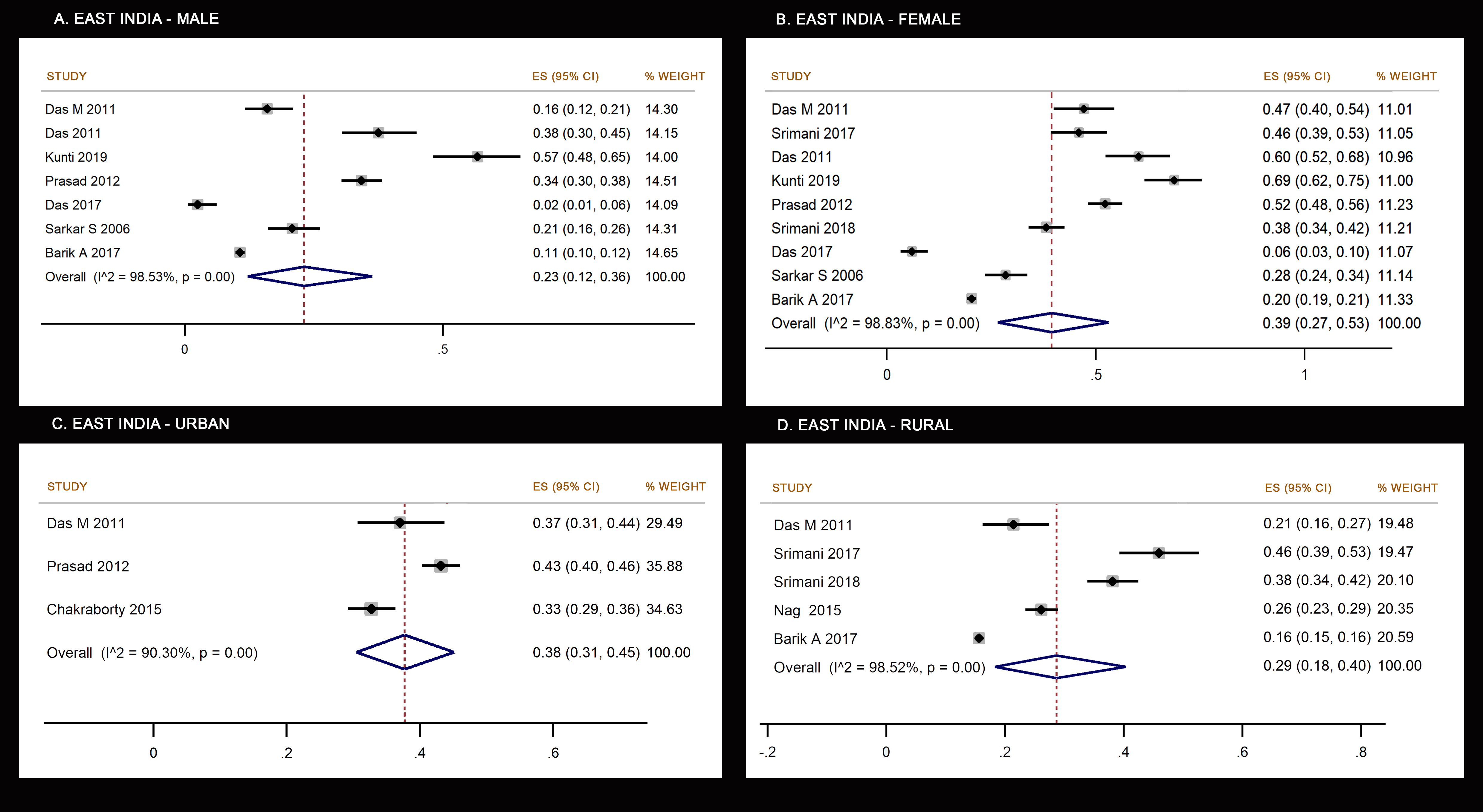

Supplement: S4 Fig — Forest plot showing the setting wise and gender wise distribution of metabolic syndrome in Eastern India a) Eastern India–Male b) Eastern India–Female c) Eastern India–Urban d) Eastern India–Rural. (TIFF) [file pone.0240971.s005.tiff]

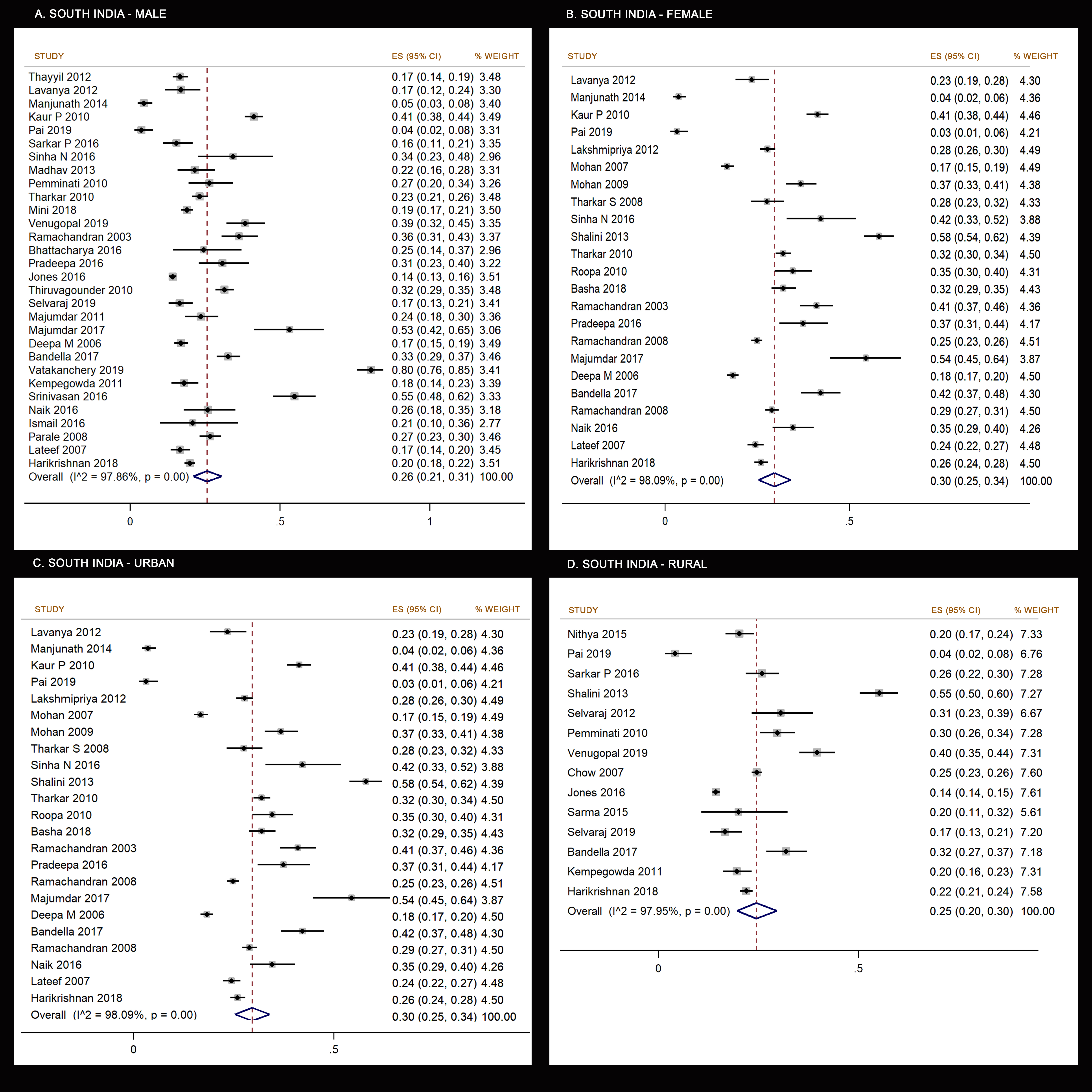

Supplement: S5 Fig — Forest plot showing the setting wise and gender wise distribution of metabolic syndrome in South India a) South India–Male b) South India–Female c) South India–Urban d) South India–Rural. (TIFF) [file pone.0240971.s006.tiff]

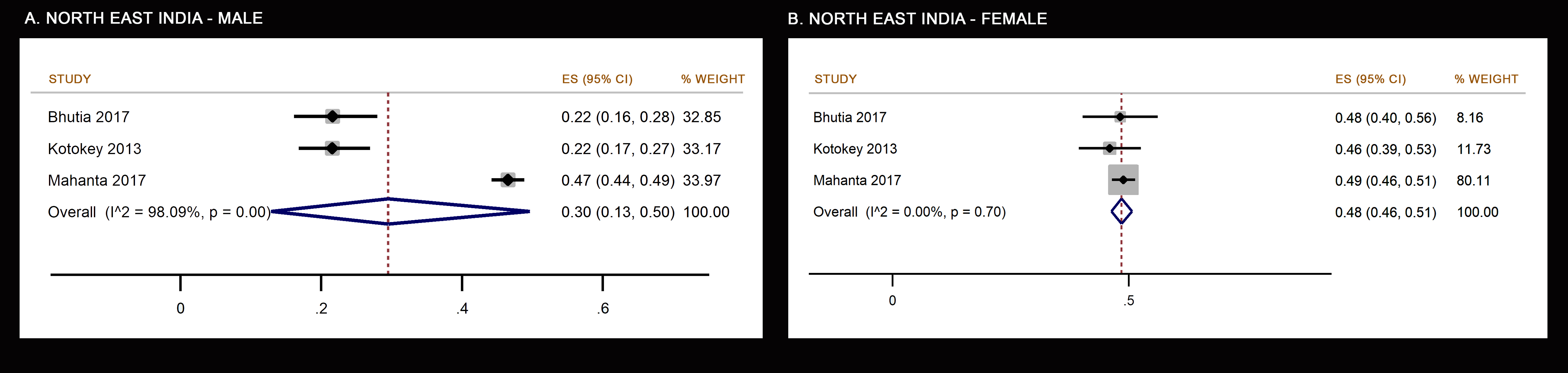

Supplement: S6 Fig — Forest plot showing the setting wise and gender wise distribution of metabolic syndrome in Northeast India a) Northeast India–Male b) Northeast India–Female. (TIFF) [file pone.0240971.s007.tiff]
