# Supplementary material for: Prevalence of metabolic syndrome among adult population in India: A systematic review and meta-analysis
Source: PLoS One. 2020 Oct 19;15(10):e0240971. doi: 10.1371/journal.pone.0240971 (PMC7571716; doi:10.1371/journal.pone.0240971)
Supplement: S1 File — (PDF) [file pone.0240971.s008.pdf]

Supplementary File 1 Search strategy

PubMed Search: ((((((((((Metabolic Syndrome) OR Syndrome) OR Insulin resistance syndrome) OR ((((Hypertension) OR high blood pressure)) AND ((Hyperlipidemia) OR lipid disorder))) OR ((((Hypertension) OR high blood pressure)) AND (((hyperglycemia) OR diabetes mellitus) OR high blood sugar))) OR ((abdominal obesity) AND ((Hypertension) OR high blood pressure))) OR ((((Hyperlipidemia) OR lipid disorder)) AND (((hyperglycemia) OR diabetes mellitus) OR high blood sugar))) OR ((abdominal obesity) AND ((Hyperlipidemia) OR lipid disorder))) OR ((abdominal obesity) AND (((hyperglycemia) OR diabetes mellitus) OR high blood sugar)))) AND prevalence) AND India

Filters applied: Observational Study, English, Humans

Time point: Inception till July 2019

Citations obtained: 172 results
